# Perioperative management of aortic stenosis in patients undergoing non-cardiac surgery

**DOI:** 10.3389/fcvm.2023.1145290

**Published:** 2023-04-06

**Authors:** Roberto A. Herrera, Mark M. Smith, William J. Mauermann, Vuyisile T. Nkomo, Sushil Allen Luis

**Affiliations:** ^1^ Department of Cardiovascular Medicine, Mayo Clinic, Rochester, MN, United States; ^2^ Department of Anesthesiology and Perioperative Medicine, Mayo Clinic, Rochester, MN, United States

**Keywords:** aortic stenosis (AS), aortic valve replacement (AVR), outcomes, non-cardiac surgery, valvular heart disease (VHD)

## Abstract

Aortic stenosis is one of the most common cardiac valve pathologies in the world and its prevalence increases with age. Although previously associated with increased perioperative mortality, more recent studies suggest that mortality rates may be decreasing. Recent guidelines suggest that major non-cardiac surgery can be performed safely in asymptomatic severe aortic stenosis patients with close hemodynamic monitoring. Among symptomatic patients, the guidelines recommend aortic valve intervention prior to major non-cardiac surgery because of a reduction in the incidence of postoperative heart failure and improved rates of long-term overall survival. This review provides a comprehensive and contemporary review of the perioperative management of patients with severe aortic valve stenosis.

## Introduction

Aortic stenosis (AS) is one of the most common cardiac valve pathologies in the world and its prevalence increases with age. A population-based study of 11,911 participants estimated the prevalence of AS to be 2.8% in patients above the age of 75 (1). Risk factors predisposing patients to AS most commonly include smoking, hypertension, and hyperlipidemia (2). A progressive calcification of valve leaflets, in conjunction with inflammation and lipid accumulation, leads to intensifying rigidity as well as decreased mobility and valve opening (3). Patients with AS also tend to develop left ventricular hypertrophy (1). Furthermore, severe AS often leads to significant symptom burden and is associated with a high mortality rate. Even in patients with asymptomatic severe AS, the probability of remaining free of cardiac death or the need for surgical aortic valve replacement is estimated to be 80% at 1 year, 63% at 2 years, and 25% at 5 years (4). Patients with severe AS are at an increased risk of developing a major adverse cardiovascular event (MACE) following major non-cardiac surgery. This disease has traditionally been associated with increased perioperative mortality, although more recent studies suggest that mortality rates may be decreasing.

## Preoperative assessment of aortic stenosis

Preoperative assessment of AS typically includes an evaluation of the degree of AS and assessment of concomitant diseases. Two significant comorbidities to consider are concomitant coronary artery disease and chronic kidney disease (CKD).

In addition to conducting a thorough history and physical examination, the degree of AS can be non-invasively assessed with transthoracic echocardiography (TTE). Echocardiography plays an important role in studying aortic valve morphology and hemodynamics, left ventricular hypertrophy, and systolic and diastolic dysfunction, as well as other valvular diseases (5). Current guidelines provide a class 1 recommendation that patients with known AS undergo perioperative TTE if no prior echocardiogram was done within 1 year or if a significant change in clinical status or physical examination has occurred since the previous evaluation, in order to implement appropriate perioperative management (6, 7). Annual progression of aortic valve stenosis is expected to result in nearly a 3 mmHg increase in the transaortic pressure gradient with a 0.3 cm^2^ decrease in the aortic valve area (8). Key hemodynamic measures provided by TTE for stenotic valvular lesions include maximum velocity, mean gradient, and valve area (9). The goal of preoperative assessment is to categorize patients at high risk during the procedure and intervene, if possible, to mitigate the risks associated with the procedure. Of note, systemic hypertension imposes additional pressure on the left ventricle and augments valve obstruction, thus resulting in decreased forward flow and potentially a lower transaortic gradient. Therefore, it is best that the gradient be measured when the patient is normotensive to avoid underestimation of the severity of valvular disease (9).

One of the most difficult concomitant disease processes to assess is coronary artery disease (CAD). The landmark PARTNER-2 Trial showed some degree of CAD present in over 60% of patients undergoing either transcatheter aortic valve replacement (TAVR) or surgical aortic valve replacement (SAVR) (10). Previous trials have estimated 30%–40% of patients with severe AS to have concomitant CAD with about half of those patients having single vessel disease. Furthermore, around a third of the patients with CAD and severe AS did not have anginal symptoms to suggest this as a comorbidity (11). The 2014 ACC/AHA guidelines state that an evaluation of CAD is warranted in combination with an electrocardiographic exercise study, stress echocardiography, nuclear imaging stress study, or coronary angiography as indicated (6). To our knowledge, there have not been any large-scale studies comparing Single Photon Emission Computed Tomography (SPECT) with coronary angiogram as part of the preoperative assessment of patients with CAD and severe AS, although smaller studies have been performed. A 2017 study found that 33 patients with severe AS who initially underwent SPECT myocardial perfusion imaging with a vasodilator and subsequent coronary angiography had a sensitivity, specificity, and diagnostic accuracy of 77%, 69%, and 73%, respectively (12).

Another important consideration prior to major non-cardiac surgery is renal function. Patients with CKD have a higher prevalence of AS and the severity of AS correlates with mortality in this population (13, 14). Five-year survival estimates in patients with severe AS and CKD were 42% compared with 67% in patients without CKD (14). To our knowledge, there are no large studies assessing the risk of new dialysis or acute kidney injury (AKI) in patients with severe AS and concomitant CKD undergoing major non-cardiac surgery. Pre-existing CKD is the most significant risk factor for the development of AKI after aortic valve replacement, and baseline estimated glomerular filtration rate (eGFR) is an important variable in preprocedural risk calculators (13). The magnitude of AKI after cardiac surgery has been shown to increase incident CKD, progression of CKD, and mortality in a graded manner (13, 15). Further studies are warranted to help guide patients in decision-making.

## Risks involved with asymptomatic and symptomatic patients with aortic stenosis undergoing major non-cardiac surgery

### Intraoperative risks and considerations

Asymptomatic patients with severe aortic stenosis without left ventricular dysfunction can reasonably pursue elective non-cardiac surgery without antecedent aortic valve intervention, although appropriate intraoperative and postoperative hemodynamic monitoring are recommended (6, 7). Patients with severe AS likely have an increased risk of MACE and mortality intraoperatively. The mechanism by which this occurs is thought to be due to hypotension and tachycardia from anesthetic agents and surgical stress leading to injurious hemodynamics. Unexpected bleeding from the surgical site can also furthercompromise hemodynamics. An unfavorable hemodynamic state can lead to decreased coronary perfusion, myocardial injury, development of arrythmias or ischemia, and death (6). In a recent retrospective cohort study assessment, intraoperative outcomes during non-emergent/non-urgent major non-cardiac surgery were evaluated in 203 patients who had undergone aortic valve replacement (AVR) previously and were compared with 288 patients who had not undergone prior AVR. They found that patients without prior AVR had significantly higher rates of red blood cell transfusions (26.4% vs. 15.8%), a higher use of Swan-Ganz monitoring (16.7% vs. 5.5%), and higher catecholamine utilization (16.7% vs. 1.5%) (16). Although urgent non-cardiac surgery is typically associated with unfavorable hemodynamics, Okuno et al. demonstrated a borderline significant association between the risk of the 30-day composite outcome associated with urgent non-cardiac surgery after TAVR (58.6% vs. 43.8%, *p* = 0.05), but this association was not significant on multivariable analysis [adjusted hazard ratio (aHR): 1.60 (95% CI: 0.94–2.73), *p* = 0.08] (17). Given the borderline *p*-values, this may reflect a lack of statistical power for the identification of increased risk among patients undergoing urgent surgery, and larger studies are required to explore this further.

### Postoperative risks

The postoperative outcomes of patients with severe AS have been well studied. An important distinction is asymptomatic compared with symptomatic AS. In a meta-analysis of nearly 30,000 participants, patients with asymptomatic AS had an increased risk of composite cardiovascular adverse events (Relative Risk (RR): 1.59, 95% CI: 1.19–2.12), without an increased risk of death (18). Among symptomatic AS patients, there was a significant increase in the risk of myocardial infarction (RR: 3.87, 95% CI: 1.31–11.46), but without significant differences in other observed outcomes (18).

In a retrospective propensity-matched case control study, 634 patients with AS were compared with 2,536 controls (19). This study evaluated patients with moderate AS (valve area 1.0 cm–1.5 cm^2^) and severe AS (valve area <1.0 cm^2^). The composite outcome of death and myocardial infarction (MI) at 30 days was worse for patients with both moderate and severe AS compared with controls. Postoperative MI at 30 days was significantly higher in the AS group than in the control group. The mortality rate was also higher at 30 days in the AS group than in the control group (2.1% vs. 1.0% *p* = 0.036) (19).

Another retrospective case control study comparing 256 patients with severe AS with 256 controls undergoing high-risk or intermediate risk non-cardiac surgery found no difference in the 30-day mortality rate. The presence of symptoms with AS (syncope, angina, or dyspnea) was associated with a higher 30-day mortality rate. Death and MACE at 30 days were very similar in asymptomatic patients compared with controls. Symptomatic patients with severe AS had a significantly higher occurrence of MACE at 30 days (28.3% vs. 8.5%, *p* ≤ 0.001). Although a trend toward increased 30-day mortality was noted in symptomatic patients, it did not reach statistical significance (9.4% vs. 3.8%, *p* = 0.097). One-year mortality was significantly higher among symptomatic patients than among controls (16.0% vs. 6.6%, *p* < 0.001), but this difference did not reach statistical significance among asymptomatic patients compared with controls (14.0% vs. 8.7%, *p* = 0.14) (20).

The most recent evaluation of postoperative risks by Luis et al. looked at the composite endpoint of MACE such as death, stroke, ST-segment myocardial infarction, non-ST-segment myocardial infarction, ventricular arrhythmias, and new or worsening heart failure at 30-day postoperation and found this to be significantly less common in the group with prior AVR compared with the non-AVR group (5.4% vs. 20.5%). Patients who underwent AVR and ultimately did not undergo non-cardiac surgery because of death or complication from the AVR were not included in this study. Most of the differences in outcomes resulted from a lower incidence of new or worsening heart failure, as defined by the treating physician's clinical assessment, in the AVR group (2.5% vs. 17.7%). When heart failure was excluded from the analysis, no difference in MACE was seen. There was also no significant difference in the death rate between the groups at 30 days. The average length of stay was 6 days in the non-AVR group compared with 4 days in the AVR group (*p* < 0.001) (16).

A small study comparing 12 patients undergoing major non-cardiac surgery with 126 patients who did not undergo surgery proposed that major non-cardiac surgery was associated with a more rapid progression of AS compared with patients with similar baseline characteristics not undergoing such surgery (21). More studies are needed to evaluate this potential risk (21).

## Considerations in predicting risk

**Figure 1:**
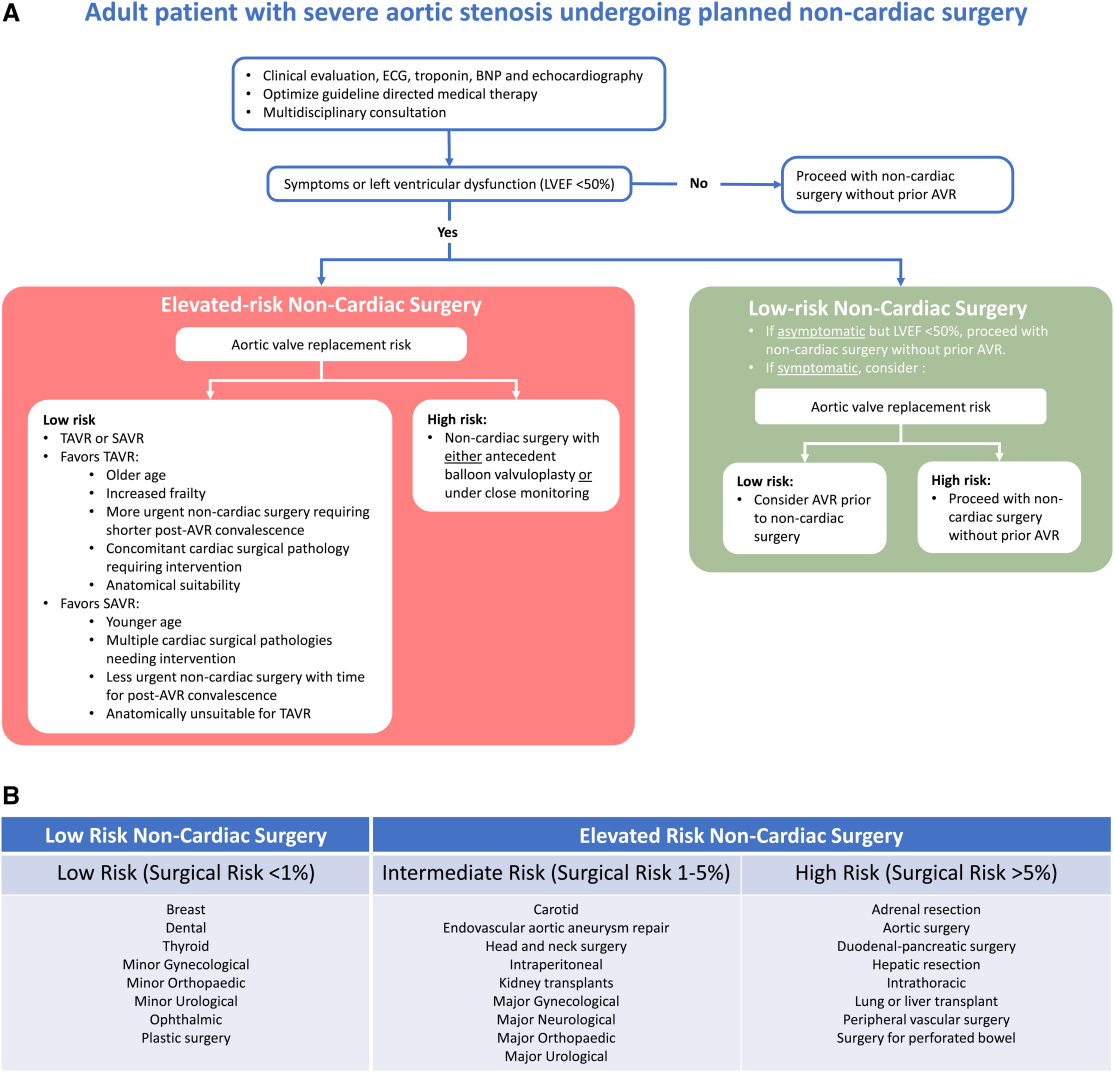
(**A**) Suggested management algorithm for patients with severe aortic valve stenosis undergoing non-cardiac surgery. (**B**) Estimated procedural risk by surgery categorized according to ACC/AHA and ESC guidelines (6, 7). AVR, aortic valve replacement; BNP, brain natriuretic peptide; ECG, electrocardiogram; LVEF, left ventricular ejection fraction; TAVR, transcatheter aortic valve replacement; SAVR, surgical aortic valve replacement.

Not only individual patient comorbidities but also the type of procedure or surgery needs to be taken into account to estimate cardiovascular morbidity and mortality for the patient. The surgical-related risk is determined by the type and length of the operation. Surgical risk estimates broadly appraise a 30-day risk of cardiovascular (CV) death, MI, and stroke on the basis of the surgical intervention proposed, without considering the patient's comorbidities (7). ESC guidelines suggest a classification of surgical risk into low (<1% surgical risk), intermediate (1%–5% surgical risk), and high risk (≥5% surgical risk), while ACC/AHA guidelines suggest categorization into low (<1% surgical risk) vs. elevated risk (≥1% surgical risk) (Figure 1) (6, 7). An observational study from the CURRENT AS Registry assessed 30-day mortality rates in 187 patients with untreated severe AS and 161 patients who had undergone AVR prior to surgery, identifying that higher surgical risk estimates incrementally increased the risk of 30-day mortality in the untreated severe AS group, although this difference was not statistically significant (low risk: 0%, intermediate risk: 4.3%, high risk: 6.6%; *p* = 0.46) (22).

The previously cited study by Tashiro et al. also evaluated the predictors of poor outcomes. They found that emergency surgery was the strongest predictor of 30-day mortality in patients with severe AS and those without. Atrial fibrillation and serum creatinine levels above 2.0 mg/dL were also predictors of death at 30 days on multivariate analysis. Higher stroke volumes, larger aortic valve areas, as well as the use of statins were associated with improved survival (20). Agarwal et al. also found that high-risk surgery, symptomatic severe AS, coexisting mitral regurgitation, and pre-existing CAD were significant predictors of 30-day mortality and postoperative MI in patients with AS undergoing major non-cardiac surgery (19). Predictors of 1-year mortality were the presence of severe AS and a left ventricular ejection fraction (LVEF) under 55% (20).

## Indications for valve intervention prior to non-emergent major non-cardiac surgery

The indications for aortic valve intervention, with either TAVR or SAVR, are outlined in the 2020 ACC/AHA Guidelines for the Management of Valvular Heart Disease. Symptomatic adults with high-gradient severe AS (stage D1) carry a class I indication (level of evidence A). Symptoms can include exertional dyspnea, angina, syncope, presyncope, or heart failure. Where symptomatology is uncertain, exercise stress testing should be considered where feasible. Additional class I indications (level of evidence B-NR) include asymptomatic patients with severe AS and LVEF (<50%), asymptomatic patients with severe AS already undergoing cardiac surgery for other operative indications, and symptomatic patients with low-flow, low-gradient severe AS with reduced or normal LVEF if the AS is thought to be the cause of symptoms (9).

Class 2a indications for intervention include asymptomatic patients with severe AS and decreased exercise tolerance or fall in systolic >10 mmHg from baseline during exercise tolerance testing, asymptomatic patients with an aortic velocity >5 m/s, asymptomatic patients and a serum B-type natriuretic peptide over three times the normal values, and asymptomatic patients with high-gradient severe AS with an increase in aortic velocity over 0.3 m/s per year. All of the class 2a indications are with the inclusion that the patient is a low surgical risk (9).

Patients who meet an indication for valve surgery prior to major non-cardiac surgery can consider aortic valve replacement. Aortic valve replacement before non-cardiac surgery is associated with a decreased incidence of worsening heart failure after non-cardiac surgery and improved overall survival without differences in 30-day survival rates, MI, ventricular arrhythmia, or stroke (16). If they are deemed high risk or ineligible for SAVR, major non-cardiac surgery can be considered with invasive hemodynamic monitoring and optimizing loading conditions. Transcatheter aortic valve intervention is also an option, as sometimes is percutaneous aortic balloon dilation (6).

Limitations of the currently available literature should be noted. The CURRENT AS registry and that by Luis et al. included patients who underwent AVR in preparation for non-cardiac surgery in addition to those who underwent antecedent AVR without this specific indication prior to non-cardiac surgery (16, 22). Noting such limitations, future randomized control trials are required to definitively address the role of antecedent AVR prior to non-cardiac surgery.

## Scenarios in which valve replacement can reasonably be declined

Aortic valve replacement may be considered in many patients with severe AS. The PARTNER trial identified that in patients who could not undergo valve replacement via a surgical approach, the 1-year mortality rate for those receiving standard therapy including aortic balloon valvuloplasty was 50.7%. The trial also noted that only five patients needed to be treated with TAVR to prevent one death in the first year in the PARTNER trial (23). Furthermore, recently published data showed a reduction in new or worsening heart failure in patients who underwent AVR prior to elevated risk non-cardiac surgery. Symptomatic patients who underwent AVR prior to elevated-risk non-cardiac surgery also had a lower 30-day mortality rate compared with those who did not receive valve replacement (16). These data suggest that ideally, patients undergoing a higher risk non-cardiac surgery should undergo antecedent AVR to minimize the risk of poor outcomes postoperatively.

There are instances in which antecedent valve replacement may not be the ideal option for the patient. Such scenarios include aggressive malignancies or other instances where non-cardiac surgery cannot be safely delayed and limited patient longevity due to comorbidities or poor quality of life. In such instances, it should be noted that although prior AVR in asymptomatic severe AS patients did reduce the incidence of new or worsening heart failure, it did not reduce the 30-day mortality rate when compared with those patients who underwent AVR prior to non-cardiac surgery (16). Hence, it is reasonable to pursue elevated-risk elective non-cardiac surgery in an asymptomatic patient with severe AS with close intraoperative and postoperative hemodynamic monitoring (6, 16). Valve replacement may not be the best option in a patient with a short life expectancy irrespective of their AS, such as a patient with metastatic cancer (16). In a patient with less than 1 year of an acceptable quality of life, a palliative care assessment may be the most appropriate (9).

## Selection of valve intervention strategy

If AVR is pursued by the patient and their provider, the choice of intervention strategy is a complex decision to take. Several components need to be considered, including the age, risks, and patient values. In intermediate-risk patients undergoing AVR, TAVR, and SAVR have been shown to have similar 2-year outcomes when looking at death or disabling stroke. When evaluating the transfemoral access cohort alone, TAVR resulted in a lower rate of death or disabling stroke than surgery. TAVR was associated with lower rates of acute kidney injury, severe bleeding, and new-onset atrial fibrillation as well as a larger postprocedural aortic valve area. On the other hand, SAVR resulted in fewer major vascular complications and less paravalvular aortic regurgitation (10).

The timing of non-cardiac surgery after AVR is another important aspect to be considered. A prospective single-center cohort study looked at the timing and risks of non-cardiac surgery after TAVR, with most of the patients undergoing intermediate-risk non-cardiac surgery (17). The study included 21.0% who underwent non-cardiac surgery within 30 days of TAVR, 25.0% between 31 days and 180 days, 23.0% between 181 days and 365 days, and 31.0% more than 1 year after TAVR (17). Timing from TAVR, urgency of surgery, and risk of surgery were not associated with an increased risk of the primary composite outcome of all-cause death, stroke, MI, and major or life-threatening bleeding at 30-day post-non-cardiac surgery. Moderate to severe patient–prosthesis mismatch and moderate to severe paravalvular regurgitation were independently associated with an increased risk of the primary outcome (17). More data are needed to further evaluate these findings and future studies are needed to compare non-cardiac surgical outcomes in patients who undergo SAVR vs. TAVR.

In high-risk patients with severe AS, TAVR, and SAVR have similar survival rates at 1 year (24). The PARTNER Trial investigators found that the incidence rate of major stroke was higher in the TAVR group than in the SAVR group, although not significant (5.1% vs. 2.4%, *p* = 0.07) (24). The surgical group had higher rates of major bleeding and new-onset atrial fibrillation. At 30 days, the TAVR group had higher rates of major vascular complications.

If a patient is a high surgical risk, disqualifying them from surgery, such as a patient with an Society of Thoracic Surgery (STS) risk score above 8% or a very frail patient, but has an acceptable quality of life with a life expectancy of more than 1 year, TAVR should be considered. If surgery is not prohibited on the basis of risk to the patient, then postoperative anticoagulation needs to be taken into consideration. A patient who has a contraindication to a vitamin K antagonist (VKA) and a patient who cannot be reasonably adherent to VKA or who declines VKA will need a bioprosthetic valve. If the patient can tolerate VKA and they are under the age of 65, a surgical approach with a mechanical valve is likely to be the most appropriate approach (9).

A bioprosthetic valve can be pursued via either a surgical approach or TAVR, but age, vascular anatomy, and life expectancy are important factors. Where both SAVR and TAVR are feasible, patient preferences must be strongly considered. In a patient needing a bioprosthetic valve without suitable vascular anatomy to pursue TAVR, SAVR is the appropriate strategy if they are below the age of 65. In patients between the ages of 65 and 80, a transfemoral approach can be considered if the valvular anatomy including annulus size and shape, degree of calcification, number of valve leaflets, and coronary ostial height are amenable to TAVR. If over the age of 80 and the valvular characteristics and vascular anatomy are amenable to a transcatheter approach, then TAVR may be preferable in light of increased age-associated frailty and comorbidities.

Aortic balloon valvuloplasty alone usually results in early symptomatic improvement, although patients receiving this intervention are at risk of developing serious acute complications such as restenosis, clinical deterioration, and severe aortic regurgitation within 6–12 months. Therefore, balloon valvuloplasty is not a substitute for AVR (9). A single-center study of 15 patients did find balloon valvuloplasty to be well tolerated and effective, with a 100% survival rate at 6 months in patients who underwent subsequent non-cardiac surgery (25). More data are needed to investigate this, although balloon valvuloplasty may serve as a short-term bridge for poor surgical and procedural candidates undergoing non-cardiac surgery, particularly if life expectancy is already short.

## Conclusion

AS is a common valvular pathology and severe disease is associated with a high mortality rate (1, 4). Preoperative evaluation when considering valve replacement includes TTE and assessment of concomitant diseases, with coronary artery disease and chronic kidney disease being two of the most important to consider (9, 10, 13, 14). Identifying if symptoms are attributed to AS is an important part of patient assessment, as patients with symptomatic severe AS are at an increased risk of poor outcomes mostly in the form of new or worsening heart failure (16, 20). Symptomatic patients may also have an increased risk of mortality if they are to undergo elevated-risk non-cardiac surgery (16, 19, 20). Routine societal guidelines should be followed in the management of aortic valve stenosis regardless of the need for non-cardiac surgery (9). Where indicated, and particularly in symptomatic patients, aortic valve replacement should be considered prior to planned non-cardiac surgery. Regardless, in certain patients, it may be reasonable to forego valve replacement prior to aortic valve replacement particularly in patients where surgery cannot be safely deferred (e.g., aggressive malignancies) or where a palliative approach is chosen in those with a life expectancy of less than 1 year of an acceptable quality of life (9, 16). Selection of valve intervention is a complex decision that must take into consideration patient age, values, preferences, ability to adhere to medications, valvular characteristics, and vascular anatomy (9, 23, 24).
